# (2*R*)-Ethyl 2-(5-bromo-2,3-dioxoindolin-1-yl)propanoate

**DOI:** 10.1107/S1600536808020588

**Published:** 2008-07-09

**Authors:** Alexander V. Kurkin, Anna A. Bernovskaya, Marina A. Yurovskaya, Victor B. Rybakov

**Affiliations:** a Department of Chemistry, Moscow State University, 119991 Moscow, Russian Federation

## Abstract

The title compound, C_13_H_12_BrNO_4_, was obtained from an optically active aniline derivative. The structure was characterized by ^1^H NMR, ^13^C NMR, MS and X-ray diffraction techniques. 86% of the atoms of the two independent mol­ecules in the asymmetric unit show non-crystallographic inversion symmetry.

## Related literature

For related structures, see: Akkurt *et al.* (2006 ▶); Miehe *et al.* (1991 ▶); Robeyns *et al.* (2007 ▶). For general background, see: Sandmeyer (1919 ▶); Silva *et al.* (2001 ▶); Spek (2003 ▶).
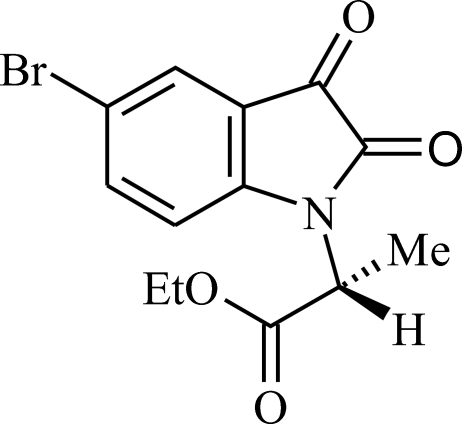


## Experimental

### 

#### Crystal data

C_13_H_12_BrNO_4_
*M* *_r_* = 326.14
Monoclinic, 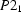

*a* = 9.7390 (13) Å
*b* = 14.355 (2) Å
*c* = 9.8361 (10) Å
β = 95.779 (9)°
*V* = 1368.1 (3) Å^3^
*Z* = 4
Cu *K*α radiation
μ = 4.20 mm^−1^
*T* = 293 (2) K
0.20 × 0.20 × 0.20 mm

#### Data collection

Enraf–Nonius CAD-4 diffractometer
Absorption correction: ψ scan (North *et al.*, 1968 ▶) *T*_min_ = 0.385, *T*_max_ = 0.432
6047 measured reflections
5502 independent reflections
3935 reflections with *I* > 2σ(*I*)
*R*_int_ = 0.020
1 standard reflection frequency: 60 min intensity decay: 2%

#### Refinement

*R*[*F* ^2^ > 2σ(*F* ^2^)] = 0.051
*wR*(*F* ^2^) = 0.140
*S* = 1.02
5502 reflections
348 parameters
1 restraint
H-atom parameters constrained
Δρ_max_ = 0.45 e Å^−3^
Δρ_min_ = −0.38 e Å^−3^
Absolute structure: Flack (1983 ▶), 2566 Friedel pairs
Flack parameter: −0.06 (3)

### 

Data collection: *CAD-4 EXPRESS* (Enraf–Nonius, 1994 ▶); cell refinement: *CAD-4 EXPRESS*; data reduction: *XCAD4* (Harms & Wocadlo, 1995 ▶); program(s) used to solve structure: *SHELXS97* (Sheldrick, 2008 ▶); program(s) used to refine structure: *SHELXL97* (Sheldrick, 2008 ▶); molecular graphics: *ORTEP-3* (Farrugia, 1997 ▶); software used to prepare material for publication: *WinGX* (Farrugia, 1999 ▶).

## Supplementary Material

Crystal structure: contains datablocks global, I. DOI: 10.1107/S1600536808020588/bt2723sup1.cif

Structure factors: contains datablocks I. DOI: 10.1107/S1600536808020588/bt2723Isup2.hkl

Additional supplementary materials:  crystallographic information; 3D view; checkCIF report

## Figures and Tables

**Fig. 1.**
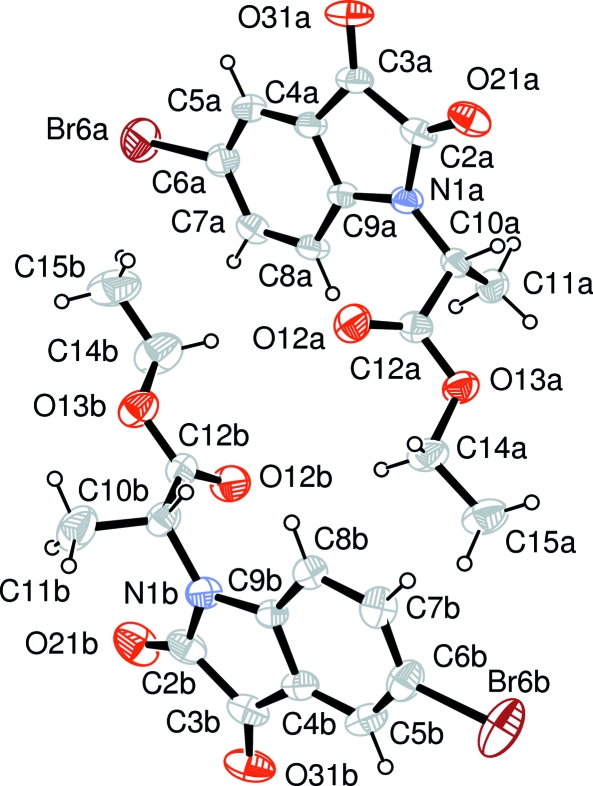
ORTEP–3 (Farrugia, 1997) plot of the molecules (A and B) of compound I with the numbering scheme. Thermal displacement ellipsoids are shown at the 30% probability level. H atoms are drawn as a small spheres of arbitrary radius.

**Fig. 2.**
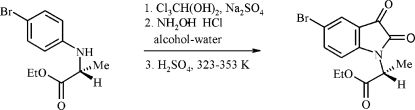
Sinthesis of ethyl (2R)–2–(5–bromisatin–1–yl)propanoate, I.

## supplementary crystallographic information

### Comment

Nowadays, much attention has been focused on isatin derivatives for their broad–spectrum biological and pharmacological activities, such as antibacterial, antiprotozoal, antifungal, antiviral, anti–HIV, anticonvulsant, antihelminthic activities, influence *CNS*, participate in metabolism and stimulate growth of plants (Silva *et al.*, 2001). There is a modern tendency to use pure enantiomers of heterocyclic compounds instead of their racemic mixtures, for example, as starting materials in preparation of pharmaceuticals. It is true for the derivatives of isatin. In this paper, we report the synthesis and crystal structure of the ethyl (2*R*)–2–(5–bromisatin–1–yl)propanoate.

The asymmetric unit of the title compound has two independent molecules (hereafter called A and B), which depicted in Fig. 1. The ADDSYM test by *PLATON* (Spek, 2003), shown a noncrystallographic inversion.

In the principle, the geometric parameters of heterobicycle are closely agree with ones in molecular structures of ethyl 2–(2,3–dioxoindolin–1–yl)acetate (Robeyns *et al.*, 2007), *N*–benzylindole–2,3–dion (*N*–benzylisatin) (Akkurt *et al.*, 2006) and 1–methyl–1*H*–indole–2,3–dione (Miehe *et al.*, 1991).

The short interatomic contacts O12*a*···C3b = 3.01Å and O12*b*···C3a = 2.98Å were found in the crystal structure.

### Experimental

We have synthesized ethyl (2*R*)–2–(5–bromisatin–1–yl)propanoate from optically active aniline by Sandmeyer method (Sandmeyer, 1919) (Fig. 2). A mixture of solutions of chloralhydrate (0.003 mol) in water (5.1 ml), a solution of ethyl (*R*)–*N*–(4–bromophenylamino)propanoate (0.0018 mol) in water (1.23 ml) with concentrated hydrochloric acid (0.26 g), a solution of hydroxylamine hydrochloride (0.0061 mol) in water (1.03 ml) and Na_2_SO_4_ (0.42 g), was stirred at reflux for 1–2 min. In addition we have used ethanol as a solvent to increase aniline solubility. The reaction mixture was cooled to r.t., extracted with CH_2_Cl_2_ and dried over anhydrous Na_2_SO_4_. The solvent was evaporated under reduced pressure to afford isonitroso–substance as brown oil (83%). The isonitroso–substance (0.0015 mol) was added to concentrate sulfuric acid (1.46 g) at 323 K so that the temperature of the reaction mixture did not exceed 343 K. The reaction mixture was stirred at 353 K for 10–15 min. The resulting mixture was cooled to r. t., diluted with cold water, extracted with CH_2_Cl_2_ and dried over anhydrous Na_2_SO_4_. The solvent was evaporated under reduced pressure. The crude product was purified by column chromatography on silica gel (eluent - ethylacetate/petroleum ether: 10/1) to afford ethyl (2*R*)–2–(5–bromisatin–1–yl)propanoate (60%) as a red solid, its enantiomeric purity determinates by HPLC with chiral stationary phase achieved 97% *ee. M*.p. 404–405 K.

The ^1^H NMR (CDCl_3_), δ, p.p.m., J (Hz): 1.15 (t, J=7.0, 3H, —CH_2_—CH_3_), 1.53 (d, J=7.1, 3H, —CH—CH_3_), 4.08–4.21 (m, 2H, —CH_2_—CH_3_), 5.15 (q, J=7.1, 1H, —CH—CH_3_), 7.12 (d, J=8.5, 1H, 7–H), 7.77 (s, 1H, 4–H), 7.85 (d, J=8.1, 1H, 6–H). ^13^C NMR (DMSO–d_6_), δ, p.p.m.: 14.19 (CH_3_), 14.42 (CH_3_), 49.55 (CH), 61.89 (CH_2_), 113.94 (CH), 115.71 (C), 119.92 (C), 127.52 (CH), 140.41 (CH), 148.95 (C), 157.70 (C?O), 169.73 (C?O), 181.87 (C?O). Mass–spectr., m/*z* (I, %): 224 [*M*^+^—CH_3_CHCO_2_*Et*], (10), 145 (2), 117 (8), 91 (36), 41 (39).

### Refinement

In the compound I hydrogen atoms bonded to C–atoms were included in calculated positions and refined as riding atoms. Calculated C—H bond lengths are in the range of 0.93–0.97 Å. For methyl H–atoms *U*_iso_ values were set equal to 1.5*U*_eq_ of the carrier atoms, for other H–atoms *U*_iso_ values were set to 1.2*U*_eq_ of the carrier atoms.

### Crystal data

**Table:**

C_13_H_12_BrNO_4_	*F*_000_ = 656
*M**_r_* = 326.14	*D*_x_ = 1.583 Mg m^−^^3^
Monoclinic, *P*2_1_	Melting point: 404.5 K
Hall symbol: P 2yb	Cu *K*α radiation λ = 1.54184 Å
*a* = 9.7390 (13) Å	Cell parameters from 25 reflections
*b* = 14.355 (2) Å	θ = 32.2–34.4º
*c* = 9.8361 (10) Å	µ = 4.20 mm^−^^1^
β = 95.779 (9)º	*T* = 293 (2) K
*V* = 1368.1 (3) Å^3^	Prism, red
*Z* = 4	0.20 × 0.20 × 0.20 mm

### Data collection

**Table:**

Enraf–Nonius CAD-4 diffractometer	*R*_int_ = 0.020
Radiation source: Fine–focus sealed tube	θ_max_ = 74.9º
Monochromator: graphite	θ_min_ = 4.5º
*T* = 293(2) K	*h* = −12→12
Non–profiled ω scans	*k* = −17→17
Absorption correction: ψ scan(North *et al.*, 1968)	*l* = −12→12
*T*_min_ = 0.385, *T*_max_ = 0.432	1 standard reflections
6047 measured reflections	every 60 min
5502 independent reflections	intensity decay: 2%
3935 reflections with *I* > 2σ(*I*)	

### Refinement

**Table:**

Refinement on *F*^2^	Hydrogen site location: inferred from neighbouring sites
Least-squares matrix: full	H-atom parameters constrained
*R*[*F*^2^ > 2σ(*F*^2^)] = 0.051	*w* = 1/[σ^2^(*F*_o_^2^) + (0.0642*P*)^2^ + 0.5869*P*] where *P* = (*F*_o_^2^ + 2*F*_c_^2^)/3
*wR*(*F*^2^) = 0.140	(Δ/σ)_max_ = 0.001
*S* = 1.02	Δρ_max_ = 0.45 e Å^−^^3^
5502 reflections	Δρ_min_ = −0.38 e Å^−^^3^
348 parameters	Extinction correction: SHELXL97 (Sheldrick, 2008), Fc^*^=kFc[1+0.001xFc^2^λ^3^/sin(2θ)]^-1/4^
1 restraint	Extinction coefficient: 0.0039 (3)
Primary atom site location: structure-invariant direct methods	Absolute structure: Flack (1983)
Secondary atom site location: difference Fourier map	Flack parameter: −0.06 (3)

### Special details

**Table:**

Experimental. Number of ψ–scan sets used was 8. The θ correction was applied. Averaged transmission function was used. No Fourier smoothing was applied.
Geometry. All s.u.'s (except the s.u. in the dihedral angle between two l.s. planes) are estimated using the full covariance matrix. The cell s.u.'s are taken into account individually in the estimation of s.u.'s in distances, angles and torsion angles; correlations between s.u.'s in cell parameters are only used when they are defined by crystal symmetry. An approximate (isotropic) treatment of cell s.u.'s is used for estimating s.u.'s involving l.s. planes.
Refinement. Refinement of *F*^2^ against ALL reflections. The weighted *R*–factor *wR* and goodness of fit *S* are based on *F*^2^, conventional *R*–factors *R* are based on *F*, with *F* set to zero for negative *F*^2^. The threshold expression of *F*^2^ > 2σ(*F*^2^) is used only for calculating *R*–factors(gt) *etc*. and is not relevant to the choice of reflections for refinement. *R*–factors based on *F*^2^ are statistically about twice as large as those based on *F*, and *R*–factors based on ALL data will be even larger.

### Fractional atomic coordinates and isotropic or equivalent isotropic displacement parameters (Å^2^)

**Table:**

	*x*	*y*	*z*	*U*_iso_*/*U*_eq_	
N1a	0.0320 (4)	0.0742 (3)	0.5522 (4)	0.0519 (9)	
C2a	0.0599 (5)	−0.0181 (3)	0.5310 (6)	0.0558 (11)	
O21a	0.1299 (4)	−0.0683 (3)	0.6073 (5)	0.0748 (11)	
C3a	−0.0156 (6)	−0.0421 (3)	0.3892 (6)	0.0599 (13)	
O31a	−0.0148 (5)	−0.1195 (2)	0.3406 (5)	0.0838 (13)	
C4a	−0.0793 (5)	0.0440 (3)	0.3390 (5)	0.0536 (12)	
C5a	−0.1571 (6)	0.0641 (4)	0.2187 (6)	0.0655 (14)	
H5a	−0.1750	0.0188	0.1517	0.079*	
C6a	−0.2075 (6)	0.1520 (4)	0.1997 (6)	0.0683 (15)	
Br6a	−0.31241 (9)	0.18369 (7)	0.03306 (8)	0.1113 (3)	
C7a	−0.1789 (6)	0.2207 (4)	0.2980 (6)	0.0603 (13)	
H7a	−0.2141	0.2804	0.2822	0.072*	
C8a	−0.1000 (5)	0.2019 (3)	0.4175 (5)	0.0542 (12)	
H8a	−0.0799	0.2482	0.4827	0.065*	
C9a	−0.0506 (5)	0.1117 (3)	0.4392 (5)	0.0468 (10)	
C10a	0.0842 (5)	0.1204 (3)	0.6760 (5)	0.0524 (11)	
H10a	0.1315	0.0724	0.7341	0.063*	
C11a	−0.0284 (6)	0.1595 (4)	0.7571 (5)	0.0663 (15)	
H11a	−0.0726	0.2112	0.7085	0.099*	
H11b	0.0119	0.1801	0.8450	0.099*	
H11c	−0.0954	0.1119	0.7687	0.099*	
C12a	0.1957 (5)	0.1904 (4)	0.6456 (5)	0.0539 (11)	
O12a	0.2353 (4)	0.2019 (3)	0.5372 (4)	0.0787 (12)	
O13a	0.2439 (4)	0.2352 (3)	0.7588 (4)	0.0654 (10)	
C14a	0.3501 (6)	0.3044 (5)	0.7423 (6)	0.0764 (17)	
H14a	0.4308	0.2749	0.7111	0.092*	
H14b	0.3160	0.3506	0.6753	0.092*	
C15A	0.3859 (7)	0.3487 (5)	0.8757 (7)	0.089 (2)	
H15A	0.3052	0.3772	0.9060	0.133*	
H15B	0.4552	0.3954	0.8675	0.133*	
H15C	0.4208	0.3026	0.9408	0.133*	
N1b	0.5010 (4)	0.5226 (3)	0.4543 (4)	0.0580 (10)	
C2b	0.4556 (6)	0.6130 (4)	0.4568 (7)	0.0662 (15)	
O21b	0.3847 (5)	0.6536 (3)	0.3690 (6)	0.0947 (15)	
C3b	0.5115 (6)	0.6509 (4)	0.5989 (7)	0.0671 (15)	
O31b	0.4928 (5)	0.7288 (3)	0.6363 (6)	0.0896 (15)	
C4b	0.5872 (5)	0.5728 (4)	0.6681 (5)	0.0567 (12)	
C5b	0.6581 (6)	0.5656 (5)	0.7945 (6)	0.0692 (15)	
H5b	0.6639	0.6155	0.8551	0.083*	
C6b	0.7207 (5)	0.4817 (5)	0.8295 (5)	0.0689 (14)	
Br6b	0.82477 (10)	0.46814 (8)	1.00249 (8)	0.1231 (4)	
C7b	0.7096 (6)	0.4070 (4)	0.7425 (6)	0.0671 (14)	
H7b	0.7517	0.3510	0.7701	0.080*	
C8b	0.6371 (5)	0.4131 (4)	0.6151 (5)	0.0553 (12)	
H8b	0.6295	0.3623	0.5560	0.066*	
C9b	0.5761 (5)	0.4980 (3)	0.5786 (5)	0.0459 (10)	
C10b	0.4649 (5)	0.4574 (4)	0.3427 (5)	0.0596 (12)	
H10b	0.5158	0.3999	0.3665	0.072*	
C11b	0.5102 (7)	0.4896 (7)	0.2087 (7)	0.106 (3)	
H11d	0.4538	0.5411	0.1747	0.159*	
H11e	0.5008	0.4394	0.1440	0.159*	
H11f	0.6050	0.5090	0.2219	0.159*	
C12b	0.3120 (5)	0.4334 (4)	0.3396 (5)	0.0559 (12)	
O12b	0.2423 (4)	0.4511 (3)	0.4281 (4)	0.0780 (11)	
O13b	0.2720 (4)	0.3850 (3)	0.2285 (4)	0.0750 (11)	
C14b	0.1338 (7)	0.3446 (7)	0.2229 (9)	0.105 (3)	
H14c	0.1250	0.3089	0.3054	0.126*	
H14d	0.0654	0.3940	0.2178	0.126*	
C15b	0.1098 (8)	0.2856 (6)	0.1066 (9)	0.114 (3)	
H15d	0.1309	0.3188	0.0265	0.171*	
H15e	0.0147	0.2668	0.0959	0.171*	
H15f	0.1676	0.2315	0.1189	0.171*	

### Atomic displacement parameters (Å^2^)

**Table:**

	*U*^11^	*U*^22^	*U*^33^	*U*^12^	*U*^13^	*U*^23^
N1a	0.059 (2)	0.0364 (19)	0.059 (2)	0.0009 (17)	−0.0007 (18)	−0.0023 (17)
C2a	0.052 (3)	0.038 (2)	0.079 (3)	−0.001 (2)	0.015 (2)	0.002 (2)
O21a	0.074 (2)	0.045 (2)	0.104 (3)	0.0080 (18)	0.006 (2)	0.008 (2)
C3a	0.065 (3)	0.041 (3)	0.075 (3)	−0.006 (2)	0.016 (3)	−0.012 (3)
O31a	0.094 (3)	0.0413 (19)	0.116 (4)	−0.0057 (19)	0.011 (3)	−0.027 (2)
C4a	0.062 (3)	0.040 (3)	0.059 (3)	−0.008 (2)	0.008 (2)	−0.008 (2)
C5a	0.076 (4)	0.056 (3)	0.064 (3)	−0.021 (3)	0.003 (3)	−0.009 (3)
C6a	0.065 (3)	0.073 (4)	0.065 (3)	−0.015 (3)	−0.003 (3)	0.000 (3)
Br6A	0.1135 (6)	0.1265 (7)	0.0846 (5)	−0.0145 (5)	−0.0355 (4)	0.0150 (5)
C7a	0.059 (3)	0.048 (3)	0.074 (3)	−0.002 (2)	0.005 (3)	0.008 (2)
C8a	0.059 (3)	0.035 (2)	0.067 (3)	−0.004 (2)	−0.002 (2)	−0.004 (2)
C9a	0.047 (2)	0.040 (2)	0.053 (2)	−0.0066 (19)	0.005 (2)	−0.005 (2)
C10a	0.059 (3)	0.042 (2)	0.054 (3)	−0.003 (2)	0.000 (2)	0.006 (2)
C11a	0.069 (3)	0.075 (4)	0.057 (3)	−0.014 (3)	0.017 (3)	−0.006 (3)
C12a	0.048 (2)	0.053 (3)	0.062 (3)	−0.002 (2)	0.009 (2)	−0.003 (3)
O12a	0.074 (2)	0.100 (3)	0.066 (2)	−0.025 (2)	0.0229 (19)	−0.011 (2)
O13a	0.062 (2)	0.073 (2)	0.061 (2)	−0.0174 (18)	0.0030 (17)	−0.0111 (18)
C14a	0.061 (3)	0.091 (4)	0.078 (4)	−0.031 (3)	0.013 (3)	−0.021 (3)
C15a	0.071 (4)	0.102 (5)	0.092 (5)	−0.023 (4)	0.006 (3)	−0.029 (4)
N1b	0.058 (2)	0.051 (2)	0.063 (3)	0.000 (2)	−0.003 (2)	−0.0029 (19)
C2b	0.055 (3)	0.050 (3)	0.093 (4)	0.003 (2)	0.004 (3)	0.010 (3)
O21b	0.077 (3)	0.078 (3)	0.125 (4)	0.009 (2)	−0.011 (3)	0.035 (3)
C3b	0.057 (3)	0.048 (3)	0.099 (4)	−0.006 (2)	0.018 (3)	−0.002 (3)
O31b	0.085 (3)	0.044 (2)	0.142 (4)	−0.002 (2)	0.022 (3)	−0.016 (2)
C4b	0.054 (3)	0.051 (3)	0.066 (3)	−0.007 (2)	0.009 (2)	−0.012 (2)
C5b	0.060 (3)	0.076 (4)	0.071 (3)	−0.011 (3)	0.003 (3)	−0.027 (3)
C6b	0.056 (3)	0.093 (4)	0.057 (3)	0.004 (3)	−0.001 (2)	−0.006 (3)
Br6b	0.1132 (6)	0.1801 (10)	0.0686 (4)	0.0217 (6)	−0.0264 (4)	−0.0147 (6)
C7b	0.058 (3)	0.077 (4)	0.066 (3)	0.010 (3)	0.005 (3)	0.003 (3)
C8b	0.056 (3)	0.055 (3)	0.055 (3)	0.000 (2)	0.005 (2)	−0.003 (2)
C9b	0.042 (2)	0.045 (2)	0.051 (2)	−0.0056 (19)	0.0017 (18)	−0.0036 (19)
C10b	0.052 (3)	0.071 (3)	0.054 (2)	−0.002 (3)	−0.003 (2)	−0.006 (3)
C11b	0.088 (5)	0.164 (8)	0.069 (4)	−0.043 (5)	0.022 (3)	−0.027 (5)
C12b	0.058 (3)	0.059 (3)	0.051 (3)	0.000 (2)	0.003 (2)	−0.001 (2)
O12b	0.072 (2)	0.084 (3)	0.082 (3)	−0.013 (2)	0.027 (2)	−0.018 (2)
O13b	0.054 (2)	0.099 (3)	0.072 (2)	−0.013 (2)	0.0042 (18)	−0.018 (2)
C14b	0.061 (4)	0.143 (7)	0.112 (6)	−0.028 (4)	0.016 (4)	−0.037 (5)
C15b	0.086 (5)	0.117 (6)	0.140 (7)	−0.035 (5)	0.017 (5)	−0.049 (6)

### Geometric parameters (Å, °)

**Table:**

N1a—C2a	1.372 (6)	N1b—C2b	1.373 (7)
N1a—C9a	1.412 (6)	N1b—C9b	1.405 (6)
N1a—C10a	1.434 (6)	N1b—C10b	1.457 (6)
C2a—O21a	1.202 (6)	C2b—O21b	1.201 (7)
C2a—C3a	1.548 (7)	C2b—C3b	1.547 (9)
C3a—O31a	1.209 (6)	C3b—O31b	1.197 (6)
C3a—C4a	1.447 (7)	C3b—C4b	1.471 (8)
C4a—C5a	1.370 (7)	C4b—C5b	1.364 (8)
C4a—C9a	1.392 (6)	C4b—C9b	1.386 (7)
C5a—C6a	1.361 (8)	C5b—C6b	1.378 (9)
C5a—H5a	0.9300	C5b—H5b	0.9300
C6a—C7a	1.390 (8)	C6b—C7b	1.369 (8)
C6a—Br6a	1.897 (6)	C6b—Br6b	1.901 (5)
C7a—C8a	1.365 (7)	C7b—C8b	1.378 (7)
C7a—H7a	0.9300	C7b—H7b	0.9300
C8a—C9a	1.391 (7)	C8b—C9b	1.388 (7)
C8a—H8a	0.9300	C8b—H8b	0.9300
C10a—C11a	1.526 (7)	C10b—C11b	1.504 (8)
C10a—C12a	1.531 (7)	C10b—C12b	1.526 (7)
C10a—H10a	0.9800	C10b—H10b	0.9800
C11a—H11a	0.9600	C11b—H11d	0.9600
C11a—H11b	0.9600	C11b—H11e	0.9600
C11a—H11c	0.9600	C11b—H11f	0.9600
C12a—O12a	1.182 (6)	C12b—O12b	1.185 (6)
C12a—O13a	1.329 (6)	C12b—O13b	1.321 (6)
O13a—C14a	1.455 (6)	O13b—C14b	1.462 (7)
C14a—C15a	1.469 (8)	C14b—C15b	1.424 (10)
C14a—H14a	0.9700	C14b—H14c	0.9700
C14a—H14b	0.9700	C14b—H14d	0.9700
C15a—H15a	0.9600	C15b—H15d	0.9600
C15a—H15b	0.9600	C15b—H15e	0.9600
C15a—H15c	0.9600	C15b—H15f	0.9600
			
C2a—N1a—C9a	110.7 (4)	C2b—N1b—C9b	111.2 (4)
C2a—N1a—C10a	121.2 (4)	C2b—N1b—C10b	124.5 (5)
C9a—N1a—C10a	128.1 (4)	C9b—N1b—C10b	124.0 (4)
O21a—C2a—N1a	126.3 (5)	O21b—C2b—N1b	127.5 (6)
O21a—C2a—C3a	128.1 (5)	O21b—C2b—C3b	127.1 (6)
N1a—C2a—C3a	105.6 (4)	N1b—C2b—C3b	105.3 (5)
O31a—C3a—C4a	132.0 (5)	O31b—C3b—C4b	130.9 (7)
O31a—C3a—C2a	122.6 (5)	O31b—C3b—C2b	123.8 (6)
C4a—C3a—C2a	105.4 (4)	C4b—C3b—C2b	105.3 (5)
C5a—C4a—C9a	121.2 (5)	C5b—C4b—C9b	121.4 (5)
C5a—C4a—C3a	131.0 (5)	C5b—C4b—C3b	131.6 (5)
C9a—C4a—C3a	107.8 (4)	C9b—C4b—C3b	107.0 (5)
C4a—C5a—C6a	118.4 (5)	C4b—C5b—C6b	117.7 (5)
C4a—C5a—H5a	120.8	C4b—C5b—H5b	121.2
C6a—C5a—H5a	120.8	C6b—C5b—H5b	121.2
C5a—C6a—C7a	121.2 (5)	C5b—C6b—C7b	121.6 (5)
C5a—C6a—Br6a	119.8 (4)	C5b—C6b—Br6b	119.7 (5)
C7a—C6a—Br6a	118.9 (4)	C7b—C6b—Br6b	118.7 (5)
C8a—C7a—C6a	121.0 (5)	C8b—C7b—C6b	121.3 (6)
C8a—C7a—H7a	119.5	C8b—C7b—H7b	119.3
C6a—C7a—H7a	119.5	C6b—C7b—H7b	119.3
C7a—C8a—C9a	118.2 (4)	C7b—C8b—C9b	117.2 (5)
C7a—C8a—H8a	120.9	C7b—C8b—H8b	121.4
C9a—C8a—H8a	120.9	C9b—C8b—H8b	121.4
C8a—C9a—C4a	120.0 (4)	C8b—C9b—C4b	120.8 (4)
C8a—C9a—N1a	129.6 (4)	C8b—C9b—N1b	128.1 (4)
C4a—C9a—N1a	110.4 (4)	C4b—C9b—N1b	111.1 (4)
N1a—C10a—C11a	113.7 (4)	N1b—C10b—C11b	113.1 (5)
N1a—C10a—C12a	109.6 (4)	N1b—C10b—C12b	108.7 (4)
C11a—C10a—C12a	115.0 (4)	C11b—C10b—C12b	115.1 (5)
N1a—C10a—H10a	105.9	N1b—C10b—H10b	106.4
C11a—C10a—H10a	105.9	C11b—C10b—H10b	106.4
C12a—C10a—H10a	105.9	C12b—C10b—H10b	106.4
C10a—C11a—H11a	109.5	C10b—C11b—H11d	109.5
C10a—C11a—H11b	109.5	C10b—C11b—H11e	109.5
H11a—C11a—H11b	109.5	H11d—C11b—H11e	109.5
C10a—C11a—H11c	109.5	C10b—C11b—H11f	109.5
H11a—C11a—H11c	109.5	H11d—C11b—H11f	109.5
H11b—C11a—H11c	109.5	H11e—C11b—H11f	109.5
O12a—C12a—O13a	124.6 (5)	O12b—C12b—O13b	125.3 (5)
O12a—C12a—C10a	124.8 (5)	O12b—C12b—C10b	124.6 (5)
O13a—C12a—C10a	110.6 (4)	O13b—C12b—C10b	110.0 (5)
C12a—O13a—C14a	115.6 (4)	C12b—O13b—C14b	115.7 (5)
C15a—C14a—O13a	107.7 (5)	C15b—C14b—O13b	110.0 (6)
C15a—C14a—H14a	110.2	C15b—C14b—H14c	109.7
O13a—C14a—H14a	110.2	O13b—C14b—H14c	109.7
C15a—C14a—H14b	110.2	C15b—C14b—H14d	109.7
O13a—C14a—H14b	110.2	O13b—C14b—H14d	109.7
H14a—C14a—H14b	108.5	H14c—C14b—H14d	108.2
C14a—C15a—H15a	109.5	C14b—C15b—H15d	109.5
C14a—C15a—H15b	109.5	C14b—C15b—H15e	109.5
H15a—C15a—H15b	109.5	H15d—C15b—H15e	109.5
C14a—C15a—H15c	109.5	C14b—C15b—H15f	109.5
H15a—C15a—H15c	109.5	H15d—C15b—H15f	109.5
H15b—C15a—H15c	109.5	H15e—C15b—H15f	109.5
			
C9a—N1a—C2a—O21a	178.3 (5)	C9b—N1b—C2b—O21b	−176.9 (6)
C10a—N1a—C2a—O21a	−1.7 (8)	C10b—N1b—C2b—O21b	−2.6 (9)
C9a—N1a—C2a—C3a	−0.9 (5)	C9b—N1b—C2b—C3b	1.4 (6)
C10a—N1a—C2a—C3a	179.0 (4)	C10b—N1b—C2b—C3b	175.7 (4)
O21a—C2a—C3a—O31a	2.6 (9)	O21b—C2b—C3b—O31b	−2.5 (10)
N1a—C2a—C3a—O31a	−178.2 (5)	N1b—C2b—C3b—O31b	179.2 (6)
O21a—C2a—C3a—C4a	−177.6 (5)	O21b—C2b—C3b—C4b	177.8 (6)
N1a—C2a—C3a—C4a	1.7 (5)	N1b—C2b—C3b—C4b	−0.5 (6)
O31a—C3a—C4a—C5a	−1.2 (11)	O31b—C3b—C4b—C5b	0.0 (11)
C2a—C3a—C4a—C5a	179.0 (6)	C2b—C3b—C4b—C5b	179.7 (6)
O31a—C3a—C4a—C9a	178.0 (6)	O31b—C3b—C4b—C9b	179.8 (6)
C2a—C3a—C4a—C9a	−1.8 (6)	C2b—C3b—C4b—C9b	−0.5 (6)
C9a—C4a—C5a—C6a	−0.7 (8)	C9b—C4b—C5b—C6b	1.3 (9)
C3a—C4a—C5a—C6a	178.4 (6)	C3b—C4b—C5b—C6b	−179.0 (6)
C4a—C5a—C6a—C7a	1.3 (9)	C4b—C5b—C6b—C7b	−1.9 (9)
C4a—C5a—C6a—Br6a	178.7 (4)	C4b—C5b—C6b—Br6b	178.5 (4)
C5a—C6a—C7a—C8a	−0.5 (9)	C5b—C6b—C7b—C8b	1.3 (9)
Br6a—C6a—C7a—C8a	−177.8 (4)	Br6b—C6b—C7b—C8b	−179.1 (4)
C6a—C7a—C8a—C9a	−1.0 (8)	C6b—C7b—C8b—C9b	0.0 (8)
C7a—C8a—C9a—C4a	1.5 (7)	C7b—C8b—C9b—C4b	−0.6 (8)
C7a—C8a—C9a—N1a	180.0 (5)	C7b—C8b—C9b—N1b	178.0 (5)
C5a—C4a—C9a—C8a	−0.7 (8)	C5b—C4b—C9b—C8b	−0.1 (8)
C3a—C4a—C9a—C8a	−180.0 (5)	C3b—C4b—C9b—C8b	−179.8 (5)
C5a—C4a—C9a—N1a	−179.4 (5)	C5b—C4b—C9b—N1b	−178.8 (5)
C3a—C4a—C9a—N1a	1.3 (6)	C3b—C4b—C9b—N1b	1.4 (6)
C2a—N1a—C9a—C8a	−178.8 (5)	C2b—N1b—C9b—C8b	179.5 (5)
C10a—N1a—C9a—C8a	1.3 (8)	C10b—N1b—C9b—C8b	5.1 (8)
C2a—N1a—C9a—C4a	−0.2 (6)	C2b—N1b—C9b—C4b	−1.8 (6)
C10a—N1a—C9a—C4a	179.9 (5)	C10b—N1b—C9b—C4b	−176.2 (5)
C2a—N1a—C10a—C11a	−119.8 (5)	C2b—N1b—C10b—C11b	60.4 (7)
C9a—N1a—C10a—C11a	60.1 (6)	C9b—N1b—C10b—C11b	−125.9 (6)
C2a—N1a—C10a—C12a	109.9 (5)	C2b—N1b—C10b—C12b	−68.7 (6)
C9a—N1a—C10a—C12a	−70.1 (6)	C9b—N1b—C10b—C12b	104.9 (5)
N1a—C10a—C12a—O12a	−2.3 (7)	N1b—C10b—C12b—O12b	−12.8 (8)
C11a—C10a—C12a—O12a	−131.8 (6)	C11b—C10b—C12b—O12b	−140.8 (7)
N1a—C10a—C12a—O13a	179.0 (4)	N1b—C10b—C12b—O13b	171.3 (4)
C11A—C10a—C12a—O13a	49.5 (6)	C11b—C10b—C12b—O13b	43.3 (7)
O12A—C12a—O13a—C14a	1.8 (8)	O12b—C12b—O13b—C14b	−5.3 (9)
C10A—C12a—O13a—C14a	−179.5 (5)	C10b—C12b—O13b—C14b	170.6 (6)
C12A—O13a—C14a—C15a	177.7 (5)	C12b—O13b—C14b—C15b	−173.2 (7)
